# Perfect Pink Rubber Veneers

**Published:** 1907-11

**Authors:** W. E. Driscoll

**Affiliations:** Braidentown, Fla.


### PERFECT PINK RUBBER VENEERS.

By W. E. Driscoll, D. D. S., Braidentown, Fla.

It would seem that since red or black rubber is so prone to show through what is desired to be artificial pink gums, that most laboratory workers have abandoned efforts to have the stronger red or black rubber placed everywhere that safety and utility requires, and we see many cases where the pins of the teeth have only a precarious hold in the weak pink rubber, or find them loose, or the all pink rubber rim broken.

What is desired so far as practicable, is a sufficient body of the stronger material faced or veneered with the more natural looking pink, but weaker material.

Were it practicable to separate the two halves of the flask, after all the needed rubber was pressed into place, examination would show any defect that might have developed. Any one of experience knows that after a flask is packed and closed with the full amount of rubber, that very annoying complications result in displaced teeth, broken casts or other risks, if opened for examination. All the annoyances I have enumerated may be overcome by the following plan: Invest the cast with teeth in place so that no part of the wax pattern for the pink rim is covered with plaster in the bottom half of the flask. Use separating fluid, and fill upper half of flask as usual. This will give a mould for the full width of the rim in the upper half of the flask. Notice the thickness of the rim as far back as the pink rubber is to extend and calculate the amount of material that will be needed at thick and thin parts, so that the right amount may be placed at the varying points of thickness of the rim, and so that no displacement may follow final pressure on the main body of material for the plate. Let the first rubber put in position be red or black in a strip near half-inch wide and fully as thick as the plate is to be at that part, and long as the pink part is to be. Pack this strip so as to cover the pins perfectly from one end of the strip to the other. The excess of rubber after covering the pins is to be fastened to plaster back of the teeth with a hot instrument so as to allow the cast in the upper half of the flask to come evenly on to the surface of the rubber and press it in place where it is to stay. Before pressing this first strip with the cast, cut three strips of pink rubber or fold a strip of three thicknesses as it comes from the dealer or factory, wide enongh to go from the rubber already packed as high up as necessary to prevent any material except pink rubber showing in the mouth. Join one edge of this strip to the red or black rubber already covering the pins of the teeth so as to extend as far, over tops of teeth as desired. Fasten upper edge of the pink rubber to the plaster with a hot instrument to help keep it from folding down where not wanted when cast is applied to mould it. Place a strip of thin wet muslin or linen over the rubber to prevent it sticking to the cast when pressed down. Now close the two halves, both being just warm enough to not burn the fingers. It will be found that the two parts may now be separated without displacing teeth or breaking casts, as would be the case if the full amount of rubber for a complete plate had been moulded instead of this small amount. Examine now to see if anything but pink rubber will be exposed to view in the mouth, and make necessary corrections.

To strengthen the rim the necessary red or black rubber may be placed above the pink where it will not be exposed to view. Beside this at thick parts of the rim the necessary amount of red or black rubber must be placed behind the pink rubber so that there may be no displacement of the pink when closing the flask the last time.

I am supposing that a reasonable adhesive grade of rubber is to be used, and that the proper amount has been ascertained by weight for the plate. Also that the plaster is dry and coated with silex.

The remainder of rubber for main body of plate should be placed as near where it is to remain while being pressed as possible.

The last precaution is to close the front part of the flask first as much as practicable, which will secure the front part from being disturbed in some degree.

This plan secures perfect pink gums and takes very little more time or work than the haphazard uncertain way that heretofore, as a rule, has resulted in disfigured artificial gums and dissatisfaction of both patient and dentist.

